# Non-response to (statin) therapy: the importance of distinguishing non-responders from non-adherers in pharmacogenetic studies

**DOI:** 10.1007/s00228-015-1994-9

**Published:** 2015-12-19

**Authors:** S. Trompet, I. Postmus, P. E. Slagboom, B. T. Heijmans, R. A. J. Smit, A. B. Maier, B. M. Buckley, N. Sattar, D. J. Stott, I. Ford, R. G. J. Westendorp, A. J. M. de Craen, J. W. Jukema

**Affiliations:** Department of Cardiology, C5-R, Leiden University Medical Center, PO Box 9600, 2300 RC Leiden, The Netherlands; Department of Gerontology and Geriatrics, Leiden University Medical Center, Leiden, The Netherlands; Section of Molecular Epidemiology, Leiden University Medical Center, Leiden, The Netherlands; Section Gerontology and Geriatrics, Department of Internal Medicine, VU Medical Center, Amsterdam, The Netherlands; Department of Pharmacology and Therapeutics, University College Cork, Cork, Ireland; BHF Glasgow Cardiovascular Research Centre, Faculty of Medicine, Glasgow, UK; Institute of Cardiovascular and Medical Sciences, Faculty of Medicine, University of Glasgow, Glasgow, UK; Robertson Center for Biostatistics, University of Glasgow, Glasgow, UK; Faculty of Health and Medical Sciences, Department of Public Health, University of Copenhagen, Copenhagen, Denmark; Durrer Center for Cardiogenetic Research, Amsterdam, The Netherlands; Interuniversity Cardiology Institute of the Netherlands, Utrecht, the Netherlands

**Keywords:** Pharmacogenetics, Adherence, Statins, Cardiovascular

## Abstract

**Purpose:**

In pharmacogenetic research, genetic variation in non-responders and high responders is compared with the aim to identify the genetic loci responsible for this variation in response. However, an important question is whether the non-responders are truly biologically non-responsive or actually non-adherent? Therefore, the aim of this study was to describe, within the PROspective Study of Pravastatin in the Elderly at Risk (PROSPER), characteristics of both non-responders and high responders of statin treatment in order to possibly discriminate non-responders from non-adherers.

**Methods:**

Baseline characteristics of non-responders to statin therapy (≤10 % LDL-C reduction) were compared with those of high responders (>40 % LDL-C reduction) through a linear regression analysis. In addition, pharmacogenetic candidate gene analysis was performed to show the effect of excluding non-responders from the analysis.

**Results:**

Non-responders to statin therapy were younger (*p* = 0.001), more often smoked (*p* < 0.001), had a higher alcohol consumption (*p* < 0.001), had lower LDL cholesterol levels (*p* < 0.001), had a lower prevalence of hypertension (*p* < 0.001), and had lower cognitive function (*p* = 0.035) compared to subjects who highly responded to pravastatin treatment. Moreover, excluding non-responders from pharmacogenetic studies yielded more robust results, as standard errors decreased.

**Conclusion:**

Our results suggest that non-responders to statin therapy are more likely to actually be non-adherers, since they have more characteristics that are viewed as indicators of high self-perceived health and low disease awareness, possibly making the subjects less adherent to study medication. We suggest that in pharmacogenetic research, extreme non-responders should be excluded to overcome the problem that non-adherence is investigated instead of non-responsiveness.

Hydroxymethyl-3-methylglutaryl coenzyme A (HMG-CoA) reductase inhibitors (statins) are the most commonly prescribed drugs for the prevention of cardiovascular disease worldwide. Statins lower plasma cholesterol levels by 30–50 % and are associated with a reduction of cardiovascular events of 20–40 % [1]. Statins are generally well tolerated and are believed to have relatively few side effects [2]. However, clinical response is highly variable and not all subjects appear to benefit from statin therapy, with roughly a third of treated patients achieving the lipid lowering goals specified in international guidelines [1]. It is uncertain whether these subjects are truly biologically unresponsive, or are actually non-adherent to the statin therapy.

Pharmacogenetic studies aim to find genetic variation that is responsible for the variable response to drug treatment. For that purpose, high responders and non-responders are often compared with the aim to identify genetic loci associated with the variation in response [ 3, 4]. Particularly in whole genome sequencing studies, only the two extreme phenotypes (i.e., the extremely good responders and the non-responders) are chosen to reduce costs and enhance efficiency [5]. However, for correct interpretation of this comparison, it is essential to ensure that non-responders have actually taken the drug and are not merely non-responders due to non-adherence.

The most suitable studies to investigate these pharmacogenetic effects are randomized controlled trials, since adherence to medication is closely monitored, for example, through questionnaires and pill count and increasingly through electronic medication monitoring devices [6]. However, these monitoring systems do not provide absolute certainty that subjects are actually adherent to their medication [7]. Non-adherers can relatively easily bypass the control mechanisms, e.g., by discarding drugs before the pill count, which may easily lead to overestimation of adherence [8]. Using data of the PROspective Study of Pravastatin in the Elderly at Risk (PROSPER) [9, 10], we describe baseline characteristics of differential responder groups to statin treatment in order to find discriminatory factors between biologically non-responders and likely non-adherers. Furthermore, we propose how to deal with the misclassification of “false” non-responders in pharmacogenetic analyses.

## Methods

We used data from the PROSPER study [ 9, 10]. In short, the PROSPER study is a prospective multicenter randomized placebo-controlled trial to assess whether treatment with 40 mg daily pravastatin diminishes the risk of major vascular events in elderly individuals. Men and women aged 70–82 years were recruited if they had pre-existing vascular disease or were at increased risk of such disease due to smoking, hypertension, or diabetes. A total number of 5804 subjects were randomly assigned to pravastatin (*n* = 2891) or placebo treatment (*n* = 2913). At baseline, a brief medical history was taken, vital signs were recorded, and a fasting venous blood sample was collected for biochemical and hematological checks and for lipoprotein quantification. In addition, a mini-mental state examination (MMSE) was conducted to test for cognitive function, and subjects with an MMSE score of 24 or lower were excluded. The protocol of the PROSPER study meets the criteria of the Declaration of Helsinki and was approved by the medical ethics committees of each participating institution. Written informed consent was obtained from all participating cohorts.

From the pravastatin users (*n* = 2891), we excluded all subjects who were withdrawn from the PROSPER study during follow-up due to refusal of study medication or did not attend the follow-up visits (*n* = 346). For the remaining subjects (*n* = 2545), response to statin treatment was defined as the percentage of achieved LDL lowering. This was calculated by dividing the difference between baseline and post-baseline (mean of measurements at months 3, 6, 12, 24, and 36) LDL levels by the baseline level and multiplying the result by 100. If data of one or more of the measurements for one individual was missing, we took the mean of the available measurements of that individual as post-statin treatment measurement. These data were available for 2519 subjects.

We then created five groups of achieved LDL lowering (≤10, 10–20, 20–30, 30–40, >40 % LDL lowering) and compared baseline characteristics between these groups. Based on clinical experience, non-responders were defined as those subjects achieving a 10 % or lower decrease in LDL cholesterol levels and high responders were defined as those subjects achieving a decrease of more than 40 %.

First, we assessed whether there were differences in baseline characteristics between the five groups of achieved LDL lowering using ANOVA. Baseline characteristics included sex, age, education, smoking, alcohol use, BMI, blood pressure, LDL cholesterol level, history of hypertension, diabetes, vascular disease, and cognitive function. We also assessed differences in baseline characteristics between the high and non-responders with the Student’s *t* test for continuous variables and the Pearson chi-square test for categorical variables.

Second, we used binary logistic regression to assess the relative risk of being a non-responder based on the clinical characteristics that were significantly different between the high and low groups in the first analysis. Continuous measurements were dichotomized based on sex-specific medians. All analyses were adjusted for age and country of origin, and where necessary additionally adjusted for sex. Third, we calculated the number of risk factors per subject and assessed the association between the number of risk factors and non-responder status with binary logistic regression analysis adjusted for age, sex, and country of origin. We were unable to calculate the sum of risk scores in seven subjects, six of whom were high responders, due to missing data.

Finally, we performed a pharmacogenetic candidate gene study to show the effect of four well-established associated SNPs with a variable response to statin therapy using four analytic strategies [11]. The four SNPs (rs2900478 (SLCO1B1), rs445925 (APOE), rs464776 (SORT1/CELSR2/PSRC1), and (rs10455872 (LPA) were extracted from the Genome Wide Association Study (GWAS) performed in the PROSPER study, named the PHASE study (the PHArmacogenetic study of Statins in the Elderly) [12]. Genotyping was performed with the Illumina 660K beadchip, and imputation of up to 2.5 million SNPs was based on the HapMap 36 build. First, we performed a linear regression analysis to investigate the effect of the four SNPs on the achieved LDL lowering (%) in the total study sample (*n* = 2272) adjusted for age, sex, country, and baseline LDL levels. The total number of subjects is lower in this genetic analysis since the GWAS has not been executed in all PROSPER subjects, because of genotyping failure or because subjects were excluded based on the GWAS quality control criteria [12]. No subjects were excluded based on phenotypic outliers. Second, we excluded the non-responders from the sample and repeated the linear regression analysis (*n* = 2167). Third, we compared the effect of the SNPs in a case-control setting where the non-responders were set as the cases and the high responders as controls by using binary logistic regression adjusted for age, sex, country, and baseline LDL levels (*n* = 669). Lastly, we performed the same analysis using the low to moderate responders (10–20 % achieved LDL lowering) as cases (*n* = 817).

## Results

Table 1 shows the baseline characteristics of the five groups of percentage LDL lowering with pravastatin treatment. There were significant differences between the groups with regard to sex, smoking status, history of hypertension, age, education, cognitive function, alcohol use, and level of LDL cholesterol. For most characteristics there was a linear trend with LDL lowering response, for alcohol use, we observed a plateau effect for LDL lowering of 30 % and lower. Moreover, when we compared the baseline characteristics of the 114 non-responders with the characteristics of the 734 high responders to pravastatin therapy, we found that subjects who did not respond to pravastatin therapy were on average 1 year younger (*p* = 0.001), more often smoked and drank more alcohol (both *p* < 0.001), had lower LDL cholesterol levels (*p* < 0.001), had lower prevalence of hypertension (*p* < 0.001), and had lower cognitive function (*p* = 0.035), compared to subjects who highly responded to pravastatin therapy. Based on pill count, we defined a non-adherer if they returned more than 18 (20 %) pills in the preceding 90 days before their study visit (mean pill count over maximum number of study visits per individual). Within the subjects that highly responded to pravastatin therapy, 99.5 % were adherent to their study medication based on pill count, whereas in the non-responders group this was reduced to 78.6 %.

**Table 1:** Association between groups of % LDL lowering to statin treatment and clinical variables

	% LDL lowering in response to pravastatin treatment					
	>40 % (*n* = 734)	30–40 % (*n* = 989)	20–30 % (*n* = 502)	10–20 % (*n* = 180)	≤10 % (*n* = 114)	*p* ANOVA
Categorical variables (*n*, %)						
Females	423 (58)	511 (52)	218 (43)	82 (46)	56 (49)	<0.001
Current smokers	126 (17)	244 (25)	151 (30)	65 (36)	54 (47)*	<0.001
History of hypertension	503 (69)	620 (63)	301 (60)	98 (54)	58 (51)*	<0.001
History of diabetes	79 (11)	104 (11)	58 (12)	16 (8)	7 (6)	0.485
History of vascular disease	335 (46)	437 (44)	228 (45)	77 (43)	46 (40)	0.809
Country						
Scotland	325 (44)	410 (42)	210 (42)	83 (46)	49 (43)	
Ireland	248 (34)	364 (37)	200 (40)	71 (39)	51 (45)	
The Netherlands	161 (22)	215 (22)	92 (18)	26 (14)	14 (12)	0.253
Continuous variables (mean, SE)						
Age (years)	75.7 (0.12)	75.3 (0.11)	75.0 (0.15)	75.1 (0.24)	74.6 (0.29)*	0.001
BMI (kg/m^2^)	26.9 (0.15)	26.8 (0.13)	26.9 (0.18)	27.1 (0.33)	26.3 (0.42)	0.433
Education (years)	15.2 (0.08)	15.3 (0.07)	15.3 (0.10)	14.5 (0.11)	15.2 (0.19)	<0.001
MMSE (points)	28.1 (0.06)	28.2 (0.05)	28.0 (0.07)	27.8 (0.12)	27.8 (0.14)*	0.010
Alcohol (units/week)	3.5 (0.29)	5.0 (0.27)	7.2 (0.47)	7.2 (0.80)	6.5 (0.90)*	<0.001
LDL cholesterol (mmol/L)	4.0 (0.03)	3.8 (0.02)	3.7 (0.03)	3.4 (0.05)	3.4 (0.08)*	<0.001
SBP (mmHg)	156.0 (0.80)	154.0 (0.70)	155.8 (0.99)	153.4 (1.59)	152.8 (2.14)	0.200
DBP (mmHg)	83.8 (0.41)	83.6 (0.36)	83.6 (0.50)	82.7 (0.83)	83.7 (1.04)	0.828

*BMI* body mass index, *MMSE* mini-mental state examination, *LDL* low-density lipoprotein, *SBP* systolic blood pressure, *DBP* diastolic blood pressure

*Significant difference between the groups of ≤10 % and >40 % LDL lowering (all *p* < 0.05)

Next, we calculated the relative risk of being a non-responder for the characteristics that significantly differed between high and non-responders with a binary logistic regression model (Table 2). The largest relative risk was found for subjects who were current smokers (OR 3.96, 95 % CI 2.60–6.03, *p* = 1.4 × 10^−10^). We also found a higher risk of being a non-responder in subjects without a history of hypertension (OR 2.01, 95 % CI 1.32–3.04, *p* = 0.001), with a lower cognitive function (OR 1.46, 95 % CI 0.97–2.20, *p* = 0.068), with higher alcohol intake (OR 1.73, 95 % CI 1.15–2.59, *p* = 0.008), and with lower LDL cholesterol levels (OR 3.14, 95 % CI 2.05–4.80, *p* = 1.3 × 10^−7^). The association between number of characteristics and response status is also shown in Table 2. Compared to subjects with none or one risk factor, the relative risk of being a non-responder gradually increased to 15.51 (95 % CI 5.83–41.27, *p* = 4.0 × 10^−8^) for subjects with five characteristics. When the summary score was included in the model as a continuous variable, the risk of being a non-responder increased with 2.04 (95 % CI 1.69–2.46, *p* = 1.1 × 10^−13^) per additional characteristic.

**Table 2:** Association between baseline characteristics and being a non-responder

	High responders (*n* = 734)	Non-responders (*n* = 114)	OR (95 % CI)^a^	*p* value
Smoking	126 (17)	54 (47)	3.96 (2.60–6.03)	1.43 × 10^−10^
No history of hypertension	231 (32)	56 (49)	2.01 (1.32–3.04)	0.001
Low MMSE	379 (52)	68 (60)	1.46 (0.97–2.20)	0.068
High alcohol	270 (37)	58 (51)	1.73 (1.15–2.59)	0.008
Low LDL cholesterol	298 (41)	78 (68)	3.14 (2.05–4.80)	1.31 × 10^−7^
Number of characteristics				
≤1	297 (41)	20 (18)	1.0 (ref)	–
2	256 (35)	26 (23)	1.71 (0.93–3.15)	0.086
3	126 (17)	36 (32)	4.77 (2.63–8.63)	2.56 × 10^−7^
4	38 (5)	20 (18)	7.26 (3.47–15.19)	1.43 × 10^−7^
5	10 (1)	11 (10)	15.51 (5.83–41.27)	3.97 × 10^−8^
Trend			2.04 (1.69–2.46)	1.13 × 10^−13^

*OR* odds ratio, *LDL* low-density lipoprotein, *SBP* systolic blood pressure, *MMSE* mini-mental state examination

^a^The OR represents the risk of being a non-responder when you are in the risk category. The continuous factors were dichotomized based on sex-specific medians. Adjusted for age and country, the analyses for smoking and hypertension were additionally adjusted for sex

We examined the association of four well-established SNPs involved in pharmacogenetics of statins using four different research strategies (Table 3). By comparing the results for analyses 1 and 2 where we investigated the effect of the four SNPs with a continuous measurement of achieved LDL lowering (%), it is shown that by exclusion of the non-responders to statin therapy, the standard errors (SE) decreases, indicating that indeed probably noise is removed from these analyses. The beta stays more or less consistent in the analyses; however, since the SE decreases, also the *p* value decreases, and all four SNPs show significant results. In the third analysis, we compared the effect of the four SNPs in a case-control setting, where the non-responders were set as the cases and the high responders as controls, by using binary logistic regression adjusted for age, sex, country, and baseline LDL levels. Surprisingly, none of the four SNPs showed a significant association with statin response as was shown by the continuous analysis in the first two research strategies. However in analysis 4, we investigated the effect of the four SNPs also in a case-control setting, by comparing the high responders (controls) with the low-moderate responders (cases), thereby again excluding the non-responders. Now, two out of the four SNPs did again show a significant association with LDL response (Table 3).

**Table 3:** Comparison of four genetic association analyses with four well-known SNPs associated with a pharmacogenetic effect of statin therapy

	Number	Beta	SE	*p* value
rs2900478 (*SLCO1B1*)				
1. All subjects	2272	0.021	0.0065	0.0014
2. Excluding non-responders	2167	0.020	0.0061	0.0008
3. High vs non-responders	669	−0.129	0.201	0.5209
4. High vs low-moderate responders	817	−0.682	0.158	0.0001
rs445925 (*APOE*)				
1. All subjects	2272	−0.022	0.0088	0.0121
2. Excluding non-responders	2167	−0.021	0.0082	0.0097
3. High vs non-responders	669	0.398	0.327	0.2236
4. High vs low-moderate responders	817	0.170	0.262	0.5165
rs646776 (*SORT1*/*CELSR2*/*PSRC1*)				
1. All subjects	2272	−0.014	0.0058	0.0129
2. Excluding non-responders	2167	−0.016	0.0054	0.0033
3. High vs non-responders	669	0.060	0.179	0.7388
4. High vs low-moderate responders	817	0.358	0.162	0.0268
rs10455872 (*LPA*)				
1. All subjects	2272	0.0351	0.0123	0.0042
2. Excluding non-responders	2167	0.0288	0.0115	0.0122
3. High vs non-responders	669	−1.424	0.790	0.0714
4. High vs low-moderate responders	817	−0.682	0.873	0.4372

Analyses 1 and 2 were performed with linear regression with achieved LDL lowering (%) as outcome, adjusted for age, sex, country, and baseline LDL levels. Analyses 3 and 4 were performed with binary logistic regression adjusted for age, sex, country, and baseline LDL levels

## Discussion

In this study, we show that non-responders to statin treatment differ from high responders with regard to baseline clinical characteristics. Non-responders were more likely to smoke, drank more alcohol, had a lower cognitive function, were less likely to have hypertension, and had lower LDL cholesterol levels. These characteristics can be considered as indicators of higher self-perceived health and lower disease awareness, indicating that non-responders are possibly less aware of the benefits of using the study medication and are therefore more likely to be non-adherent rather than biologically unresponsive. Moreover, we show that exclusion of the non-responders in pharmacogenetic analyses yields more robust results, as the standard errors decreased after exclusion and *p* values remained significant. These results indicate that pharmacogenetic studies that compare extreme phenotypes might be at least partially biased by the phenomenon of some, perhaps many, non-adherers probably being misclassified as non-responders.

Few studies have investigated differences between non-responders and high responders of statin therapy [13–16]. These showed that characteristics that are indicators of better self-perceived health like age, the number of comorbidities, and diet habits are different between high and non-responders and are therefore more likely to be indicators of non-adherence [17, 18]. However, we cannot rule out the possibility that these characteristics truly determine whether a subject responds biologically differently to statin therapy. For example, having higher LDL cholesterol baseline levels might be associated with greater response to statin treatment simply because a greater absolute, but also relative, change is possible, and also for example by making healthy lifestyle changes on top of statin treatment. Therefore it is still not certain whether this variable can help us to discriminate between non-responders and non-adherers. This argument holds also true for smoking and gender. For both characteristics, we can think of plausible reasons that these are related to the amount of adherence, but we cannot rule out the possibility of a true pharmacological effect between these characteristics and statin treatment. However, in various subgroup analyses within the PROSPER study and in the existing literature, we found no evidence of an interaction between any of the clinical characteristics and statin response [10].

To draw more definite conclusions whether a non-responder is a true non-adherer, the plasma level of the medication should be determined. Unfortunately, we have not measured this, but even if we had, this would unfortunately not solve our problem since a patient could easily restart medication just prior to the plasma sample, while not taking the medication on other days. The second best is pill count, although also not considered highly reliable, especially not in willingly non-compliant (possibility of discarding pills just prior the pill count). Nevertheless we did keep track of the number of pills used by the participants by a provisional pill count. Based on pill count, we defined a non-adherer if they returned more than 18 (20 %) pills in the preceding 90 days before their study visit (mean pill count over maximum number of study visits per individual). Within the subjects that highly responded to pravastatin therapy, 99.5 % were adherent to their study medication based on pill count, whereas in the non-responders group, this was reduced to 78.6 %. However, since multiple studies have shown that pill count is likely not a valid instrument to measure true adherence [7, 8], we cannot draw definite conclusions from these numbers.

In many pharmacogenetic studies, non-responders are compared to high responders to investigate which genetic variants are responsible for this difference in response [5]. Although we believe that by using this design both statistical power and efficiency are maximized, there is the possibility of unintentionally investigating the non-adherent phenotype. Hence, instead of finding genetic variation responsible for the variation in response to therapy, genetic variation for adherence is assessed. Therefore, we assessed the impact of four different research strategies while performing pharmacogenetic research. Our results suggest that by excluding non-responders, the noise of any possible non-adherence is reduced, as the standard errors decreased, which cannot be the result of a larger sample size. Moreover, through the use of a case-control design for optimal efficiency, we demonstrated that by using the non-responders as cases none of the four SNPs were significantly associated whereas by using low-moderate responders, two out of the four SNPs demonstrated a significant association.

Therefore, based on these results, our suggestion is that in pharmacogenetic research, instead of comparing the extreme phenotypes (high versus non-responders), other research strategies should be undertaken to find the genetic variation responsible for the difference in response to (statin) therapy. We propose three different strategies that may be followed to diminish the likelihood of investigating non-adherence instead of non-responsiveness. First, all subjects should be investigated using the full range of responsiveness as a continuous phenotype. This way, possible non-adherers among the extremely non-responsive cases will exert limited influence on the results. The second proposed strategy is to exclude non-responsive subjects to be sure that the non-adherers are excluded from the analysis. The third, the most sophisticated, strategy is to use a propensity score, based on various clinical characteristics associated with non-adherence, to match high responders to non-responders when taking only the extremes into a pharmacogenetic study for efficiency. This last analysis will exclude any possible confounding from non-adherence from the study. Unfortunately, we could not perform such analysis in our data due to low statistical power.

In conclusion, pharmacogenetic studies that investigate the difference between high and non-responders are almost certainly in part investigating the non-adherent phenotype, since non-responders have clinical characteristics that coincide with high self-perceived health and low-disease awareness which are also very common in non-adherers. Other strategies, as proposed herein, should be used to investigate the relation between genetic variation and responsiveness to (statin) treatment.
